# The Preparation of a Challenging Superconductor Nb_3_Al by Exploiting Nano Effect

**DOI:** 10.3390/molecules28186455

**Published:** 2023-09-06

**Authors:** Chengkai Luan, Xiyue Cheng, Xiuping Gao, Jürgen Köhler, Shuiquan Deng

**Affiliations:** 1 Fujian Science & Technology Innovation Laboratory for Optoelectronic Information of China, Fuzhou 350108, China; 2 College of Chemistry and Materials Science, Fujian Normal University, Fuzhou 350007, China; 3 State Key Laboratory of Structural Chemistry, Fujian Institute of Research on the Structure of Matter, Chinese Academy of Sciences, Fuzhou 350002, China; 4 Max Planck-Institute for Solid State Research, Heisenbergstr. 1, 70569 Stuttgart, Germany

**Keywords:** Nb_3_Al, surface energy, nano effect, first principle

## Abstract

The Nb_3_Al superconductor with excellent physical and working properties is one of the most promising materials in high-magnetic-field applications. However, it is difficult to prepare high-quality Nb_3_Al with a desired superconducting transition temperature (*T*_c_) because of its narrow phase formation area at high temperatures (>1940 °C). This work reports a method to prepare stoichiometric Nb_3_Al powder samples at a relatively low temperature (1400 °C) by exploiting the nano effect of Nb particles with pretreatment of Nb powder under H_2_/Ar atmosphere. The obtained Nb_3_Al samples exhibit high *T*_c_’s of ~16.8K. Based on density functional theory (DFT) calculations and statistical mechanics analysis, the crucial role of quantum effect in leading to the success of the preparation method was studied. A new measure of surface energy (MSE) of a model particle is introduced to study its size and face dependence. A rapid convergence of the MSE with respect to the size indicates a quick approach to the solid limit, while the face dependence of MSE reveals a liquid-like behavior. The surface effect and quantum fluctuation of the Nb_n_ clusters explain the success of the preparation method.

## 1. Introduction

Among the A15 superconductors, i.e., the A_3_B intermetallic compounds of Cr_3_Si structure type with A being a transition element, Nb_3_Al has the best figure of merit regarding its critical properties such as the smaller sensitivity to stress [1,2], higher superconducting transformation temperature (*T*_c_ = 18.9 K) [3], critical current density (*J*_c_ = 1000 A/mm^2^, in 16 T, 4.2 K) [4], critical magnetic field (Bc_2_ = 32 T at 4.2 K) [5], an irradiation resistance comparable to Nb_3_Sn [6], etc. For these advantages, Nb_3_Al was considered a potential superconducting material used in an ITER (International Thermonuclear Experimental Reactor) or DEMO (demonstration power station), high-energy particle accelerators, gigahertz-class nuclear magnetic resonance (NMR) analysis, and other high-magnetic-field applications [5,7,8].

In the past few years, many attempts have been made to prepare optimal Nb_3_Al superconducting materials. However, the synthesis of pure Nb_3_Al is not straightforward because of the high melting point of Nb, the narrow phase width of Nb_3_Al, and a peritectic decomposition at 1940 °C, as shown in the Al-Nb phase diagram in Figure S1. The so-called low-temperature approaches are involved in the technologies of JR (jelly roll) [9,10], RIT (rod-in-tube) [11], CCE (clad-chip extrusion) [12], the most widely used MA (mechanical alloying) [13,14], etc. The *T*_c_ of samples obtained using these methods is far from the theoretical value, and *J*_c_ decreases rapidly in high fields under low temperatures [15]. The high-energy ball milling is a method of the MA technique widely used in recent years. However, the factors of ball milling time, ball milling temperature, ball-to-powder weight ratio, and the type of balls should be investigated many times for a batch of samples, and the impurity from equipment is unavoidable [13,16]. A reaction temperature above ~1800 °C is required for laser or electron beam irradiation [17,18] and RHQT [4,19] (rapid heating, quenching, and transformation) techniques. These approaches can normally produce Nb_3_Al samples with good high-field properties. However, besides the high energy costs and rather sophisticated devices for these methods, the preparation process is difficult to control. For laser or electron beam irradiation techniques, high temperatures always bring about grain coarsening and reduction in *J*_c_ at low magnetic fields [18]. RHQT is a relatively mature method for preparing Nb_3_Al superconducting materials, and it can produce hundreds of meters long Nb_3_Al wire [4,15]. However, according to the TEM observations by Kikuchi et al. [19], many stacking faults exist in Nb_3_Al prepared by RHQT, which causes a compositional deviation from stoichiometry. Owing to the above reasons, a low-consumption and simple method is needed for the preparation of superconducting Nb_3_Al with good performance (*T*_c_).

For the development of a better approach for the synthesis of Nb_3_Al, we recalled the fact that hydrogen exhibits high diffusivity, high solubility, and good permeability in group 5 (VB) elements (V, Nb, and Ta) [20,21,22]. For example, Nb powder in the H_2_ atmosphere is typically utilized for hydrogen storage [23,24]. Based on the first-principles calculations, Rao et al. [25,26] found that H atoms preferably located in tetrahedral interstitial sites expanding them. Moreover, it is found that one Nb vacancy is capable of trapping six H atoms, and the formation of a gas vacancy pore assists the growth of the sintered powder-specific surface [27]. These facts indicate that H atoms can obtain access to the Nb lattice, leading to enlarged lattice parameters and enhanced chemical activity [28]. Moreover, the formation of niobium hydrides [29,30,31] embrittles the Nb powder and leads to finer grain sizes. The hydrogenation of metals, followed by grinding and dehydrogenation, is a common method for preparing highly reactive fine powder samples of metals, e.g., for rare earth elements [32]. Li et al. [33,34,35] used hydrogen to treat their Nb_3_Al powder sample obtained through arc melting or spark plasma sintering. Dolukhanyan et al. [36] prepared a nonstoichiometric hydride NbH_1.23_ and used it to prepare Nb_3_A1 powder.

In this work, hydrogenation is used in a kind of pretreatment process for increasing the specific surface of Nb. Using such a reactive Nb source, stoichiometric Nb_3_Al products with *T*_c_ = 16.8 K have been successfully prepared at temperatures below 1400 °C. This method is simple, clean, and suitable for commercial large-scale production. For a better understanding of the success of this method, we calculated the quantitative increase in the surface energy of Nb particles due to the reduction in their grain sizes by using the first-principles methods and statistical mechanics analysis. The obtained results can explain the experimental findings that the active Nb particles are an essential factor for solving the serious separation problem of Nb/Al reagents due to the high evaporation rate of elemental Al.

## 2. Results and Discussion

### 2.1. Experimental

#### 2.1.1. Preparation of Reactive Nb Samples

**Figure 1:**
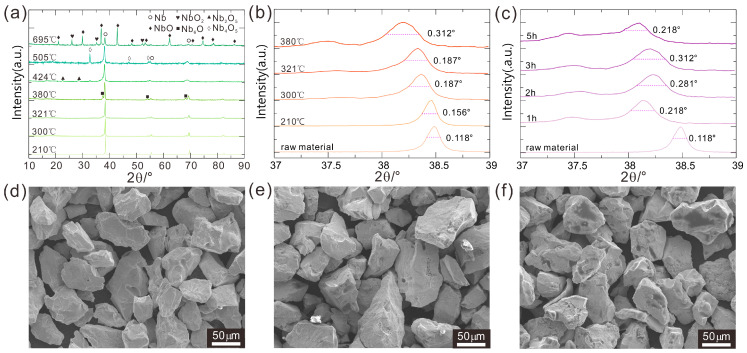
Powder XRD patterns of Nb_p_ samples (**a**) treated at various temperatures for 3 h; (**b**) treated under various temperatures for 3 h with 2θ from 37° to 39°; (**c**) sintered under 380 °C with reaction time (1 h~5 h). SEM image of (**d**) Nb raw material and T-3 h (T = (**e**) 321 °C; (**f**) 380 °C).

Nb_c_ powders have been treated under a H_2_ atmosphere at temperatures ranging from 210 °C to 695 °C and were studied by using powder X-ray diffraction; see Figure 1a. All pretreatment experiments were carried out under vacuum conditions. However, due to the less fine gas tightness of the furnace for the test experiments, contact with small amounts of air could not be avoided. At 210 °C, only the peaks from Nb can be seen, while weak peaks belonging to Nb_6_O can be found from 300 °C to 380 °C. The amount of Nb_6_O increases with increasing temperature until 424 °C, and in addition peaks from several other niobium oxides with higher oxidation states including Nb_2_O_5_, Nb_4_O_5_, etc., occur simultaneously. At even higher temperatures, more of the Nb powder reacts with O_2_ to form larger amounts of NbO and NbO_2_. For the above reason, the latter pretreatments of Nb_c_ powders were all performed below 380 °C. As Nb_6_O can easily be reduced by H_2_ at the subsequent experiments in contrast to the other niobium oxides, samples pretreated below 380 °C are acceptable. Moreover, no niobium hydrides were observed from our current powder XRD results. Comparing the SEM image of the Nb_c_ raw material and the sample pretreated at 380 °C (Figure 1d), uneven surfaces are found in the pretreated samples (Figure 1e,f). These roughened surfaces are probably caused by the Nb/H interaction and can lead to an increase in the surface area effectively, which is deemed to be important for the subsequent reaction with Al powders.

The grain size in the Nb samples prepared at various temperatures was studied through analyses of the full-width half maximum (FWHM) of the strongest diffraction peaks under uniform test conditions (Figure 1b). According to the Scherrer formula [37], the wider the FWHM, the smaller the grain size. From room temperature to 380 °C, the FWHM increased gradually from 0.118° to 0.312°, indicating that the finest grain size is obtained at 380 °C. Moreover, the lowering of the Bragg diffraction angles of the main peaks from room temperature to 380 °C is observed, corresponding to enlarged lattice parameters of Nb. The decrease in the grain size is positively related to the enlarged lattice parameters of Nb, which may be caused by both the increased temperature and the Nb/H reaction in which H atoms can interact with the Nb lattice in a very complicated way [25,26]. From the above analysis, 380 °C is deemed to be the best pretreatment temperature for Nb powders under an H_2_ atmosphere.

Similar analyses are applied to study the effect of different reaction times. The FWHM of the strongest diffraction peaks for the samples sintered under 380 °C with reaction times of 0 h~5 h and cooling in the furnace under a H_2_:Ar = 5%:95% atmosphere are shown in Figure 1c. The FWHM was found to increase to 0.312° at 3 h followed by a small decrease to 0.218° at 5 h, indicating that the grain size reaches the maximum at 3 h. As a result, samples sintered at ~380 °C with a holding time of 3 h under a mixture of hydrogen and argon atmosphere with 5% H_2_ appear optimal for the pretreatment of Nb_c_ powder.

#### 2.1.2. Synthesis of Nb_3_Al

**Figure 2:**
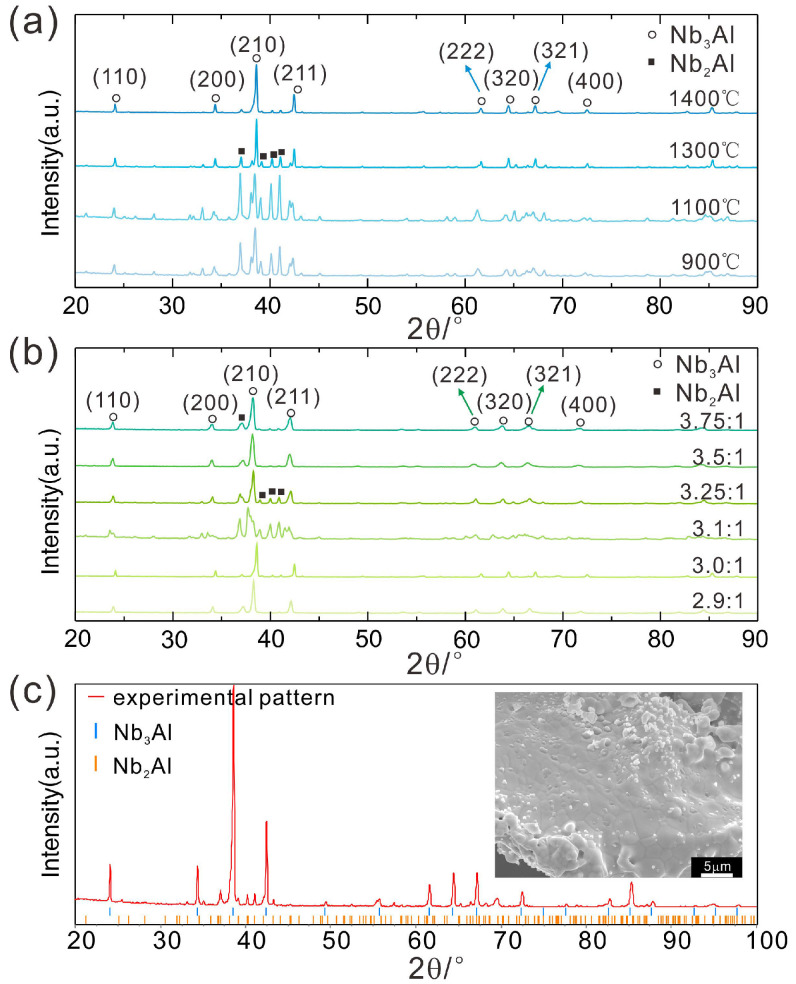
XRD patterns of Nb_3_Al samples (**a**) prepared at different temperatures for 10 h and (**b**) at 1400 °C for 10 h with different Nb_p_:Al ratios; (**c**) indexing pattern of the sample (1400 °C-10 h-Nb_p_:Al = 3.0:1) together with an SEM image of the sample as inset.

In the next step, we studied the preparation process of the target product Nb_3_Al using NbH_x_ as the starting material; see Figure 2. This can be carried out because the H_2_ adsorbed on the Nb surface or embedded into the bulk Nb can easily be removed from Nb at temperatures above 827 °C [34,38]. Furthermore, it is important to mention that the H_2_ absorption into Nb can also be remarkably reduced by the addition of Al to Nb [39].

As can be seen from Figure 2, Nb_3_Al and Nb_2_Al can both be produced under different conditions. Nb_3_Al crystallizes in the cubic space group *Pm*$\bar{3}$*n* with unit cell parameter a = 5.19 Å. Nb_2_Al crystallizes in the tetragonal space group *P*4_2_/*mnm* with unit cell parameters a = 9.94 Å, c = 5.16 Å. In both structures, the Nb and Al atoms form a certain kind of dense packing. It should be noticed that the metric of the unit cells of these intermetallic phases are related to one another in so far as a(Nb_2_Al) is approximately 2 × a(Nb_3_Al) and c(Nb_2_Al) is approximately a(Nb_3_Al). Therefore, the XRD powder patterns of Nb_2_Al (PDF 03-065-1680) and Nb_3_Al (PDF 03-065-4923) exhibit no significant differences, especially concerning the positions of the peaks.

Therefore, we determined the Nb_3_Al/Nb_2_Al ratios of the products according to the intensity of the third highest peak of the Nb_3_Al pattern around 24.5°, as shown in Figure 2a. The characteristic features of the Nb_2_Al pattern disappear gradually with increasing temperature. At a reaction temperature of 1400 °C, the target product Nb_3_Al is the main component. Obviously, Nb_3_Al can always be obtained as the main phase when sintering slightly below 1400 °C for 10 h, which holds for different ratios of Nb_p_:Al from 2.9 to 3.75:1 (Figure 2b). Among such samples, the quality of the sample with Nb_p_:Al = 3.0:1 is the best (Figure 2c, as the other samples contain more Nb_2_Al. The reaction time of 16 h is so long that, due to the penetration of small amounts of air, nitrides are formed below 1400 °C (see SI Figure S2b), and the formation of Nb_3_Al is retarded significantly or prevented [40]. When the reaction time is only 6 h, the reaction is insufficient (Figure S2a). We also tried to decrease the reaction temperature to 1350 °C and increase the sintering time to 20 h and 21 h (Figure S3), and the main phase Nb_3_Al can also be obtained when the ratio of Nb_p_:Al is 3:1. However, the quality of the samples is improved compared to the ones sintered below 1400 °C with a reaction time of 10 h. In addition, the results of the 1350 °C at 6 h series are similar to the ones at 1400 °C at 6 h. In these cases, the XRD patterns of the samples show more characteristics of Nb_2_Al, and the reaction is obviously insufficient. It should be noted that no niobium oxide was detected in any of the samples mentioned above, which means that Nb_6_O in Nb_p_ was restored or reacted during the synthesis of Nb_3_Al.

The microstructure and the elemental composition of the as-prepared Nb_3_Al samples were analyzed using SEM and EDS. As can be seen from Figures S4 and S5, the grain size of the sample sintered at 1300 °C is significantly smaller than that of the sample sintered at 1400 °C. The measured molar ratios of the elements of Nb and Al corresponding to the nominal composition (Nb_p_:Al = 2.9:1, 3.0:1, 3.5:1, 3.75:1) are 79.07:20.93 (~3.78:1), 73.69:26.31 (~2.8:1), 78.36:21.64 (~3.63:1), and 81.95:18.05 (4.5:1), respectively. There are two possible reasons for this significant difference. On the one hand, the total amount of the sample is relatively small, and the initial proportion of Nb and Al cannot be well controlled due to the huge difference in the proportion and atomic mass of Nb and Al. On the other hand, the EDS (Figure S6) measured elemental compositions are local results. The result for the sample with nominal composition Nb_p_:Al = 3.0:1 agrees best with the XRD measurement of the sample, in which the Nb_3_Al is the main component and contains only a small amount of Nb_2_Al.

**Figure 3:**
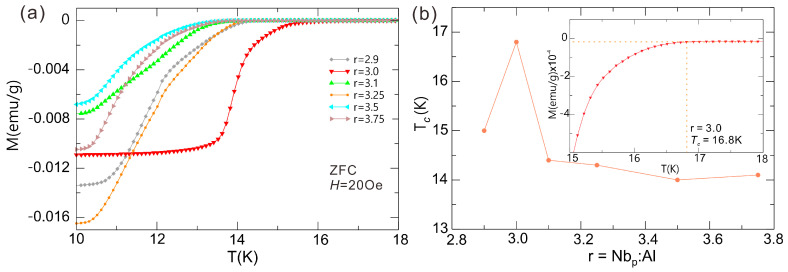
(**a**) Temperature dependence of the magnetization curves for samples sintered at 1400 °C for 10 h with different r (Nb_p_:Al); (**b**) the *T*_c_–r curves for samples sintered at 1400 °C for 10 h (the inset shows M-T curve for the sample with r = 3.0).

All Nb_3_Al samples with various nominal molar ratios r (Nb_p_:Al) sintered under 1400 °C for 10 h become superconducting; see Figure 3a. The transition temperatures *T*_c_ increase with increasing Al content in the range of 19~23 mol%, (Figure 3b), in agreement with the results obtained by Abe et al. [41]. The *T*_c_ dependence on the Al content was also confirmed by Zhang et al., though the conditions and other results are quite different [13].

The Nb:Al molar ratio of Nb_3_Al samples reported by Abe et al. [41] is lower than 3:1, which may be due to planar faults. Planar faults within the {100} layers would compensate for deviations from stoichiometry because none of the {100} layers in the A15 structure have the stoichiometric composition [41]. The A15 phases reported by Abe et al. can correspond well to the Nb-Al phase diagram, in contrast to the work by Zhang et al. [13]. This may be due to the formation of a Nb/Al solid solution after ball grinding in Zhang’s work, in which the synthesis mechanism is inconsistent with the phase diagram; see Figure S1. Although the conditions in our work correspond to the formation of a mixture of Nb_3_Al + Nb_2_Al, our results could not have been expected from the phase diagram, possibly caused by the increasing specific surface of Nb. The sample, which was sintered under 1400 °C for 10 h with r = 3.0 (25 mol% Al), has a relatively high transition temperature *T*_c_ = 16.8 K (inset illustration). Nevertheless, the boundary is different compared to the experiments of Abe et al. [41] and Zhang et al. [13]. However, the value of *T*_c_ (16.8 K) is comparable to that of Nb_3_Al samples prepared by using the RHQT (16.8 K ~ 17.3 K) method [4] and higher than the transition temperatures of Nb_3_Al samples synthesized by using the MA method (15.8 K) [13,42].

Unlike other pretreatment methods, which increase the specific surface area of Nb and Al at the same time, the pretreatment method in this paper increased only the specific surface area of Nb. Al melts completely due to its low melting point in the subsequent process. On the one hand, the reaction temperature of 1400 °C has lower requirements for synthesis equipment compared with other high-temperature technologies [18,19]. On the other hand, the sample (1400 °C-10 h-r = 3.0) prepared by using the method introduced in this paper has a higher *T*_c_ compared to the samples sintered below 1000 °C. Moreover, with this method, impurities such as Cu from Cu cans or iron from grinding balls will not be introduced into the target phase. Based on the advantages described above, the method in this work requires low consumption and is simple while yielding samples with relatively high *T*_c_. It is also suitable for the large-scale preparation of the Nb_3_Al superconducting materials.

### 2.2. Theoretical Section

It is well known that solid–solid reactions are generally much slower than liquid–liquid or liquid–solid reactions. The main reason for this is that the atoms in a solid are localized at some specific sites, which hinders both the mutual diffusion of the reagent atoms and the rearrangement of the diffused atoms into the new configurations of the products. In addition, it may also lead to inhomogeneity, phase separation, and a reacted front-layer, obstructing further reactions. For the superconducting Nb_3_Al phase, it becomes even more challenging for the reasons mentioned above and in the introduction. The key to a low-temperature synthesis of Nb_3_Al lies, in all cases, in the nano-scale contacts between the reagents before the actual reaction takes place, which creates similar reaction interfaces between the reagent particles comparable to the situation in liquid–liquid reactions. On the other hand, with the reduction in the particle size, the quantum effects become more pronounced, which enhances the diffusion through the tunneling effect, increases the surface reactivity [43], reduces the melting point [44], etc.

The calculation of ${\tilde{E}}_{bulk}$ as shown in Equation (3) or Equation (4) is straightforward. As the primitive unit cell of the $Im\bar{3}m$ structure contains only two Nb atoms, the calculated total energy is $E_{bulk}^{2}$= −20.140521 eV, thus resulting in a value of ${\tilde{E}}_{bulk}$ as −10.070261 eV. It is obvious that this value contains no surface contribution due to the periodic boundary conditions for any solids with translational symmetry. Therefore, ${\tilde{E}}_{bulk}$ is chosen as a reference point as shown in Equation (3) or Equation (4) to calculate an MSE.

**Figure 4:**
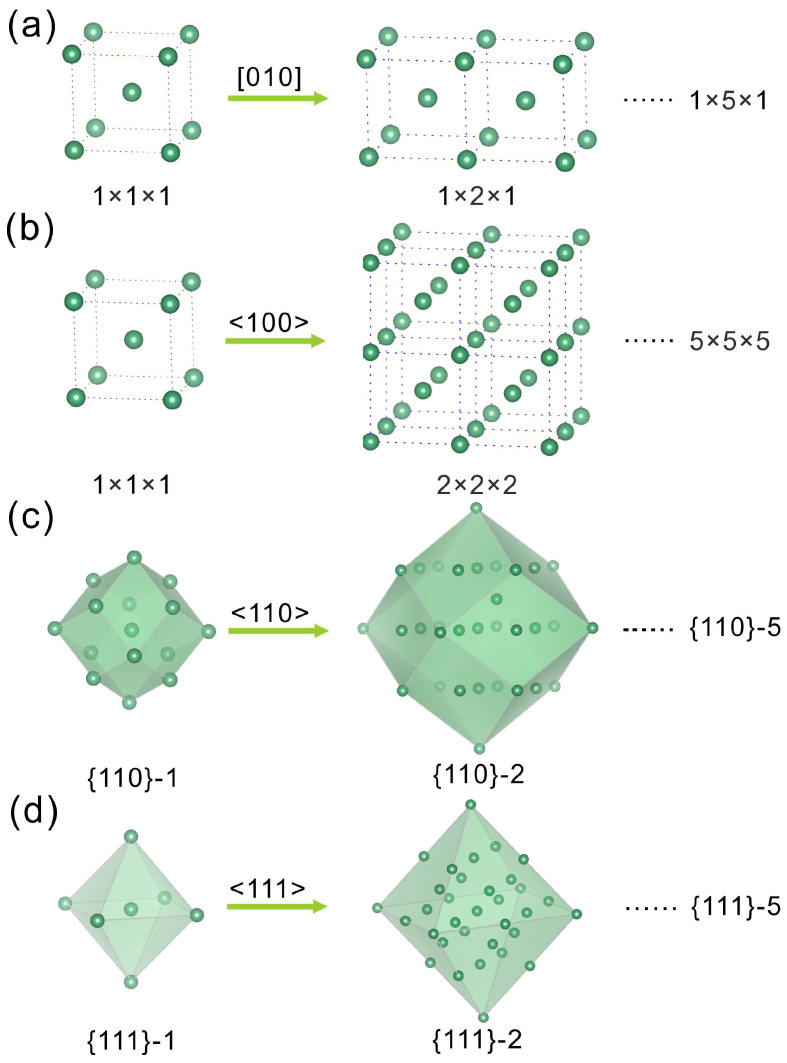
Structure of Nb units used for calculations of surface energies: (**a**) Chain model along [010] direction; (**b**) Cubic model expanded along <100> equivalent directions; (**c**) Rhombic dodecahedron model expanded along <110> directions; (**d**) Octahedron model expanded along <111> directions.

**Figure 5:**
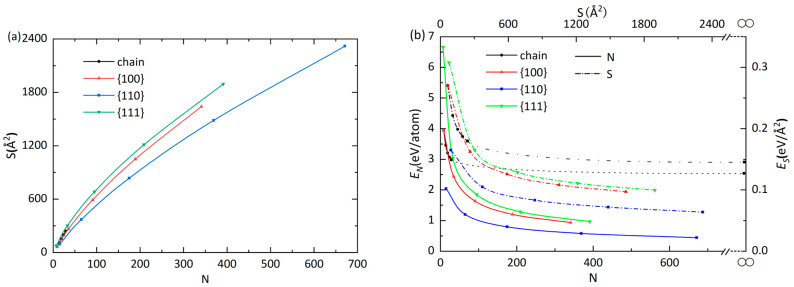
(**a**) The four curves showing the relation between MSE and the number of atoms in each type of model; (**b**) The curves of total surface area vs. number of atoms for the four types of models.

In order to explain the success of our preparation method, we used the first-principles method to calculate the MSEs of the designed Nb nanoparticle models (Figure 4). Furthermore, the face, orientation, and size dependence of MSE are also investigated. For a comparison between the new MSE, $E_{N}$, introduced in Equation (4), and the traditional MSE, $E_{S}$, defined in Equation (3), we also calculated $E_{S}$ for each model particle. In Figure 5a, we show the relations between the number of atoms in a model particle and the corresponding geometric surface area. The results indicate clearly that the geometric area *S* is approximately proportional to *N* in a wide range, and it increases monotonically with *N*. Either of them can serve as a measure of the particle size for the same material, though they have different units. This property implies that the replacement of 2*S* by *N* will not change the trend of the surface energy on the particle size, as is clear from Figure 5b. The $E_{N}$ decreases with the increase in *N* of a model particle in each of the four families. As a limit case for all Nb_n_ models, we also calculated $E_{N}$, 9.3882952 eV, for a single Nb atom, i.e., *N* = 1. As expected, it has the maximum “surface energy”. The results can easily be understood because with an increase in the particle size, the relative amount of surface atoms drops rapidly and, thus, the “surface energy” gauged by MSE ($E_{N}$ or $E_{S}$) declines accordingly, while in the current case, the single atom is of both a surface and inner character. These results explain why a smaller particle is more reactive than a big one.

However, as one can see from Figure 5b, the calculated MSE barely changes when *N* exceeds ~700 or *S* exceeds ~2300$\;{Å}^{2}$, corresponding to particles with a radius as small as ~1.35 nm. The rapid convergence of the calculated MSE on the particle size may be amended by factors such as temperature, structure instability due to various perturbations, etc., which were not considered in the first-principles calculations. As can be seen from S5 in SI, we used several hundred atoms in our models, which is far smaller than the Avogadro number. According to statistical mechanics for a canonical ensemble, the total energy of an *N*-atom system has a fluctuation due to the quantum nature and thermal effect, as follows [45,46,47].

(1) ∆EE~1N

where *E* is the mean value of the total energy; ∆E is the fluctuation around the mean value of the *N*-atom cluster defined by

(2) $${\left(∆E\right)}^{2}=\sum_{r}w_{r}{\left(E_{r}-E\right)}^{2}$$

where *r* indicates a quantum state of a cluster and $w_{r}$ is the probability of realizing this state. As can be seen clearly from Equation (1), when *N* becomes small, ∆EE becomes very large, which destabilizes the cluster and increases its reactivity dramatically. This factor continues to play a role in the rather flat tail parts of the curves in Figure 5b until *N* is large enough to be close to the Avogadro number. The resulting instability manifests itself not only in the high reactivity of a nanoparticle but also in the high tendency towards self-congregation as observed in many nanoparticle systems [43]. This instability can be driven by heat or even by zero-point energy.

Furthermore, from Figure 5b, one can clearly see that the four families of models have different MSEs for the same number of atoms in the cluster, where the last point of the chain model represents the infinite limit of that model. At this limit, the contributions from the two end surfaces can be neglected; thus, the MSE, $E_{N}$, can be approximated by that of a periodic model along the [010] direction. The revealed order of $E_{N}^{\left[010\right]}\;$> $E_{N}^{\left\{111\right\}}\;$> $E_{N}^{\left\{100\right\}}$ > $E_{N}^{\left\{110\right\}}$ for an *N* larger than 33 shows their differences in surface energy. As can be seen from Figure 5a, this order is consistent with the order of their surface area, i.e., $S_{\left[010\right]}$ > $S_{\left\{111\right\}}$ > $S_{\left\{100\right\}}$ > $S_{\left\{110\right\}}$. This result indicates that a shape with a minimum surface area is also favored by a Nb_n_ nanoparticle to lower its surface energy as a liquid drop does. Such behavior and the low melting point [43] of nanoparticles show some similarities between nanoparticle materials and liquid, which is an interesting problem worthy of further study.

The tails of all curves shown in Figure 5b exhibit a very flat feature in the region of nano-length scale, which seems to indicate a very fast convergence of the surface energy with the particle size. This result shows that the rapid drop of the MSE curves at the atomic length-scale region has nothing to do with the nano-effect exploited in preparing the Nb_3_Al superconductors. The largest diameter of our models, {110}-5, is ~3.3 nm, which is in the same order as the particle sizes obtained by using the high-energy ball mill machine [48]. Therefore, it is the tail part of the MSE curves that is relevant for the preparation of Nb_3_Al. The seemingly tiny change in MSE with the particle size does not mean the MSE is unimportant. For example, the calculated difference in $E_{N}$ between {110}-4 and {110}-5, ~0.14 eV, is already quite large for one atom, and, as can be seen from Figure 5b, this difference becomes smaller with the increase in particle size. If we approximately let only one vibrational degree of freedom be free for a surface atom (the other two inside the cluster are assumed to be frozen), then according to the energy equipartition theorem [47], the energy of this vibrational mode [49] can be estimated as ~*kT* (*k*, Boltzmann constant). At the lowest sintering temperature, 1173 K, of our experiment (see Section 3.1), the energy for one vibrational degree of freedom amounts to ~0.10 eV, a value close to the above-mentioned value of $∆E_{N}$, indicating that the vibrational energy can drive the fluctuation of the total energy or the number of atoms of a nano-cluster through the tunneling or thermal effect. These fluctuations enhance the diffusion of Nb atoms and make their reaction with Al much easier.

The rapid flattening of the MSE curves as shown in Figure 5b reveals a fast convergence of the surface energy with respect to the number of atoms of a Nb_n_ cluster, which indicates a solid behavior of larger Nb_n_ particles. An interesting issue would be the difference in the convergence speed for different transition metal clusters, which may be tightly connected to the difference in their chemical reactivity. This problem is worth studying in the future.

## 3. Materials and Methods

### 3.1. Synthesis

Highly reactive Nb powder samples (Nb_p_) as starting materials for the synthesis of Nb_3_Al were prepared from commercial niobium (Nb_c_) powder (−325 mesh, 99.99%, Alfa Aesar, Haverhill, MA, USA) under an atmosphere of hydrogen and argon mixture with 5% hydrogen (volume concentration). The pretreatment temperature of Nb_c_ was optimized by using the golden section method at 210 °C~695 °C from 1 h to 5 h. Mixtures of Nb_p_ powder and Al powder (−325 mesh, 99.5%, Alfa Aesar) with molar ratios varying from 2.80:1 to 3.75:1 were carefully ground for 0.5 h and pressed into pellets under Ar in a glove box. The pellets were wrapped with niobium foil, put into quartz glass tubes, and then heated in a tubular furnace under an atmosphere of argon with 5% hydrogen (volume concentration). The samples were sintered at 900 °C~1400 °C for 6 h~21 h.

### 3.2. Characterization

All products were characterized by using the powder X-ray diffraction (XRD, Panalytical, X’Pert Pro MPD) method with Cu-Kα radiation. The microstructure and the elemental composition of samples were investigated through scanning electron microscopy (SEM) and X-ray energy-dispersive spectroscopy (EDS) (JEOL, JSM-7001F, Akishima, Tokyo). The temperature dependence of the magnetization was measured in a 20 Oe magnetic field using a commercial SQUID magnetometer (MPMS, Quantum Design, San Diego, CA, USA). All of the M–T curves were measured over a temperature range of 10–30 K under a zero-field cooling (ZFC) mode.

### 3.3. Computational Details

The first-principles calculations were performed by using the Vienna ab initio simulation package (VASP) [50,51]. The exchange–correlation potential was calculated at the generalized gradient approximation (GGA) [52] level within the Perdew–Burke–Ernzerhof (PBE) exchange–correlation functional. The 3×3×3 (for bulk phase and all models with the total number of atoms ≤ 209; see Supporting information (SI) S5) or 1×1×1 *k*-mesh (for models having a number of atoms > 209) was used to sample the first Brillouin zones for all integrations in the momentum space. The PAW-PBE potentials [53] and the cut-off energy of 350 eV for plane wave expansions were used throughout this work. The *k*-point grids and cut-off energy were determined based on our convergence tests. In this work, the $Im\bar{3}m$ space group with a cell parameter a= 3.306 Å for the structure of bulk Nb was used [54]. To calculate the surface energy of a designed particle model (Figure 4), a 3D periodic lattice of each particle model was built with each particle separated by a 15 Å thick vacuum layer along each of the three crystal axes. As shown in Figure 4, four groups of particle models were prepared (see S5 in SI for details); each group contains five models with different sizes.

Roughly speaking, the surface of an object is characteristic of an inner side with strong interactions and an outer side without interactions. Taking this into account, surface energy is often calculated by using the following formula based on the first-principles calculations [55,56,57]:

(3) $$E_{S}=\frac{E_{slab}^{N}-N\cdot {\tilde{E}}_{bulk}}{2S}$$

where $E_{slab}^{N}$ denotes the total energy of a slab model with N atoms and two surfaces of area S for each. ${\tilde{E}}_{bulk}$ is the chemical potential of an atom in a bulk solid, and thus $E_{bulk}^{N}=N\cdot {\tilde{E}}_{bulk}$ represents the total energy of *N* atoms in a bulk solid without a surface. At the atomic length scale, it is difficult to define a “surface” as in the macroscopic case because the atomic space is not continuous and superficial atoms may not lie exactly on the same geometric plane; thus, concave/convex geometric surfaces may appear if one uses all superficial atoms in order to define a surface (e.g., see ref. [57]). However, in the practical application of Equation (3), one always uses the outermost atoms to determine a plane area S, which obviously does not reflect the nature of a rugged surface. Mathematically, the problem here involves a rigorous definition of a measure for the “area” [45] of an unsmooth curved surface spanned by a finite number of points (atoms). On the other hand, the calculated value of $E_{slab}^{N}$ depends also, to some extent, on the thickness of the designed slab. Considering the above reasons, the quantity $E_{S}$ calculated from Equation (3) should be understood as a measure of the surface energy (MSE).

The problem of MSE becomes more complicated in the case of a particle because a particle has many more surfaces than a slab (Figure 4). Furthermore, when the particle size reduces to the length scale of several nanometres, it becomes difficult to differentiate the inner bulk atoms and the surface atoms in physics (Figure 4). A recent experiment [58] reveals that nanoflakes with a thickness of several tens of nanometers show mainly surface characteristics, which indicates that an actual surface has a thickness involving atoms about one nanometer below a geometric surface, thus posing a challenge to the theoretical definition of a surface. These problems invalidate the definition of MSE at the small size limit for a particle model. For this reason, we introduce Equation (4) as follows to calculate the normalized energy needed to crop an *N*-atom particle from a bulk solid:

(4) $$E_{N}=\frac{E_{model}^{N}-N\cdot {\tilde{E}}_{bulk}}{N}$$

where $E_{model}^{N}$ is the total energy of a particle model with *N* atoms and the other symbols have the same meaning as in Equation (3). $E_{N}$ differs from $E_{S}$ in Equation (3) only in the normalization factor in the denominators. For this reason, we also call $E_{N}$ an MSE, though it has a different unit with $E_{N}$. The advantage is that it eliminates the definition problem of surface area in Equation (3). Nevertheless, in both cases, the cropping process leaves some bonds of the outermost atoms and interactions of some inner atoms unrealized, which are the origin of surface energy. Obviously, such an incompleteness of bonding/interactions can be used to explain the high reactivity of nanoparticles. It should be pointed out that a similar equation as Equation (4) was used in ref. [59] to describe the average binding energy per atom instead of surface energy.

## 4. Conclusions

High-quality samples of Nb_3_Al were prepared by using a new method. XRD experiments clearly demonstrate that the pretreatment of Nb powder in the Ar-H_2_ atmosphere can increase the specific surface areas of Nb particles, which is crucial for the preparation of Nb_3_Al. Superconducting Nb_3_Al samples with a *T*_c_ of 16.8 K have been synthesized successfully using this method. By introducing a new measure of surface energy (MSE), we have pointed out the challenges for a theoretical definition of surface energy. The main difficulty originates from the definition of the surface itself; on the one hand, the atoms on a crystal surface plane of a cluster are not necessarily equivalent. On the other hand, some inner atoms also make essential contributions to the MSE. These facts indicate that a crystal surface plane is only a mathematical simplification of an actual physical surface, which, in general, has a thickness ranging from 0.1 to 10 nanometers and is much more complicated. By using DFT calculations, we have studied the size and face dependence of MSE, which saturates at ~1.4 nm. This fact explains the difficulties of the reaction between Nb + Al. However, it is not clear whether the discovered rapid convergence of MSE applies to other metal particles. The quantum fluctuation of the Nb_n_ clusters has complemented the explanation given by the calculated MSE. The revealed minimization phenomenon of the MSE for the surface of a Nb_n_ nanoparticle revealed a liquid-like property. Our approach is a promising guideline for improving the large-scale preparations of superconducting Nb_3_Al samples.

## Data Availability

The data presented in this study are available in the article and Supplementary Materials.

## Supplementary Materials

The following supporting information can be downloaded at: https://www.mdpi.com/article/10.3390/molecules28186455/s1. The details of X-ray powder diffraction of the samples, SEM images of the samples, crystal data of Nb, and calculation results are in the supplementary material. Ref. [60] is cited in the Supplementary Materials.
