# Supplementary material for: The Preparation of a Challenging Superconductor Nb3Al by Exploiting Nano Effect
Source: Molecules. 2023 Sep 6;28(18):6455. doi: 10.3390/molecules28186455 (PMC10538086; doi:10.3390/molecules28186455)
Supplement: Supplementary file 1 [file molecules-28-06455-s001.zip › molecules-2569235-supplementary.pdf]

Supporting information

for

The Preparation of a Challenging Superconductor Nb_3Al by Exploiting Nano Effect

Chengkai Luan [1,2,3], Xiyue Cheng [1,3], Xiuping Gao [1,3], Jürgen Köhler [3,4] and Shuiquan Deng [1,3,*]

1 Fujian Science & Technology Innovation Laboratory for Optoelectronic Information of China, Fuzhou 350108, China; ckluan@fjirsm.ac.cn (C.L.); xycheng@fjirsm.ac.cn (X.C.); gaoxiuping@fjirsm.ac.cn (X.G.)
2 College of Chemistry and Materials Science, Fujian Normal University, Fuzhou 350007, China
3 State Key Laboratory of Structural Chemistry, Fujian Institute of Research on the Structure of Matter, Chinese Academy of Sciences, Fuzhou 350002, China; j.koehler@fkf.mpg.de
4 Max Planck-Institute for Solid State Research, Heisenbergstr. 1, 70569 Stuttgart, Germany
* Correspondence: sdeng@fjirsm.ac.cn

CONTENTS

S1. Phase diagram of the binary system Al-Nb

S2. X-ray powder diffraction of the samples

S3. SEM images of the samples

S4. Crystal data of Nb

S5. Particle Models and Calculated Results

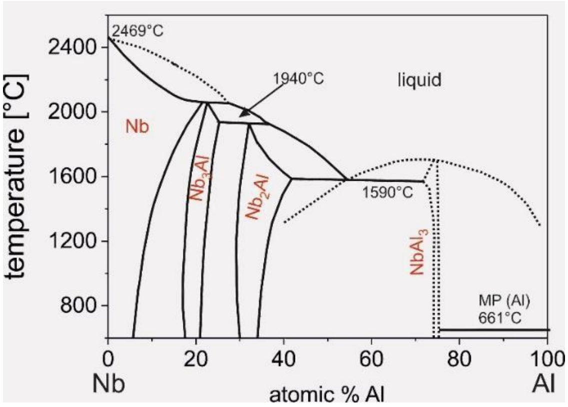

Figure S1 Reproduction of the Nb Al phase diagram according to ref. [1].

S2. X-ray powder diffraction of Nb_3Al samples

XRD patterns of Nb_3Al samples were plotted in **Figure S2-S3**. In **Figure S2**, the samples were sintered at 1400℃ with ratios of Nb_p:Al ranging from 2.8:1 to 3.6:1. The reaction times were 6h and 16h for the two series((a) and (b)). In **Figure S3**, the samples were sintered at 1350℃with ratios of Nb_p:Al ranging from 2.8:1 to 3.2:1. Here, the reaction times were 6h, 20h and 21h, respectively. All samples were sintered under a mixture of hydrogen and argon atmosphere with 5% hydrogen concentration.

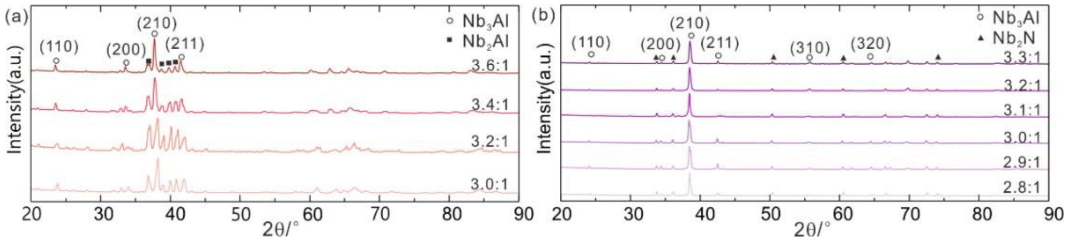

Figure S2 XRD patterns of samples prepared at 1400 ℃ with various sintering time and compositions (a) 6h (Nb_p:Al = 3.0:1, 3.2:1, 3.4:1, 3.6:1); (b) 16h (Nb_p:Al = 2.8:1, 2.9:1, 3.0:1, 3.1:1, 3.2:1, 3.3:1).

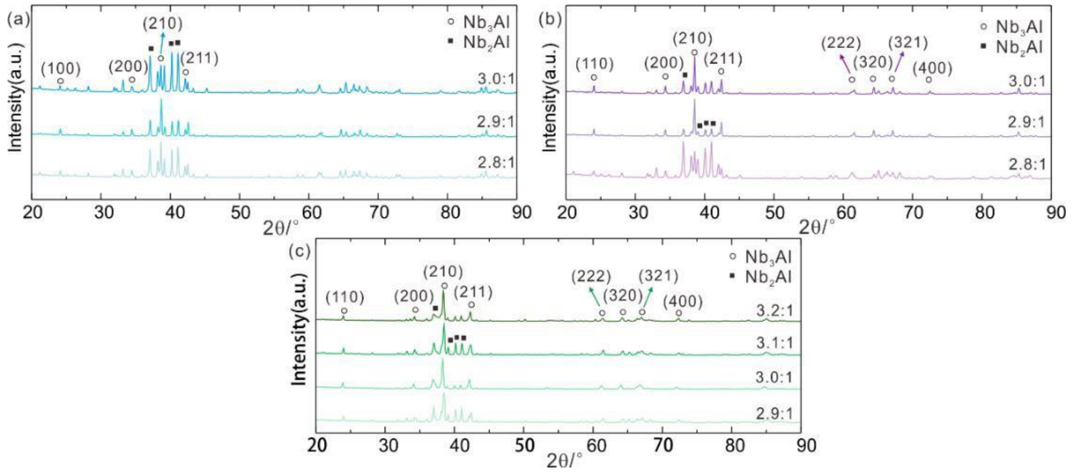

Figure S3 XRD patterns of samples prepared at 1350 ℃ with various sintering time and compositions (a) 6h (Nb_p:Al = 2.8:1, 2.9:1, 3.0:1); (b) 20h (Nb_p:Al = 2.8:1, 2.9:1, 3.0:1); (c) 21h (Nb_p:Al = 2.9:1, 3.0:1, 3.1:1, 3.2:1).

S3. SEM images of Nb_3Al samples

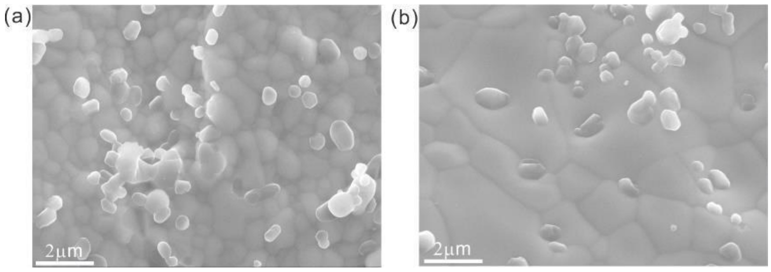

Figure S4 SEM images of Nb_3Al samples sintered at (a) 1300℃ and (b) 1400℃ for 10h with Nb_p:Al = 3.0:1

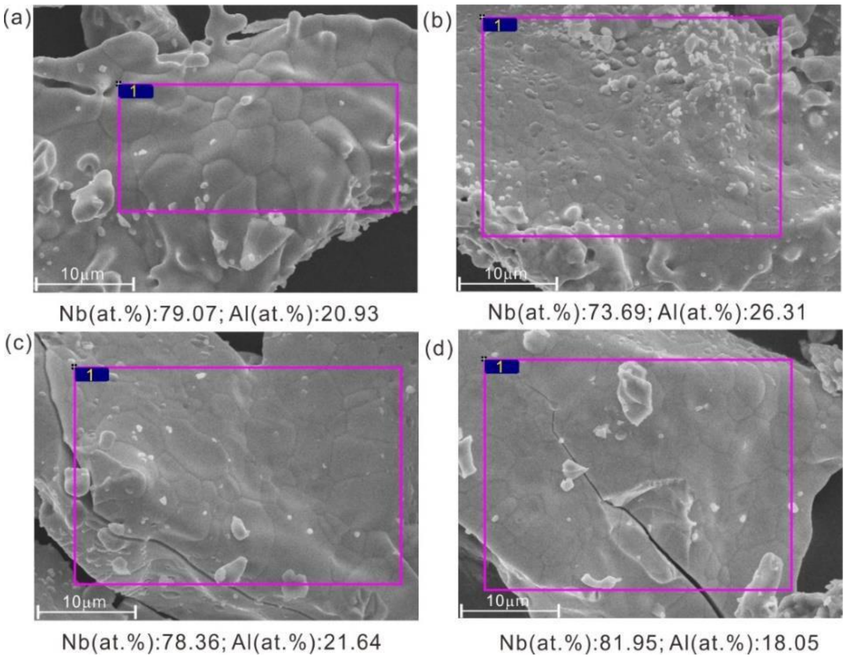

Figure S5 SEM images of Nb_3Al samples sintered at 1400°C for 10h with Nb_p: Al= (a) 2.9:1; (b)3.0:1; (c)3.5:1; (d) 3.75:1

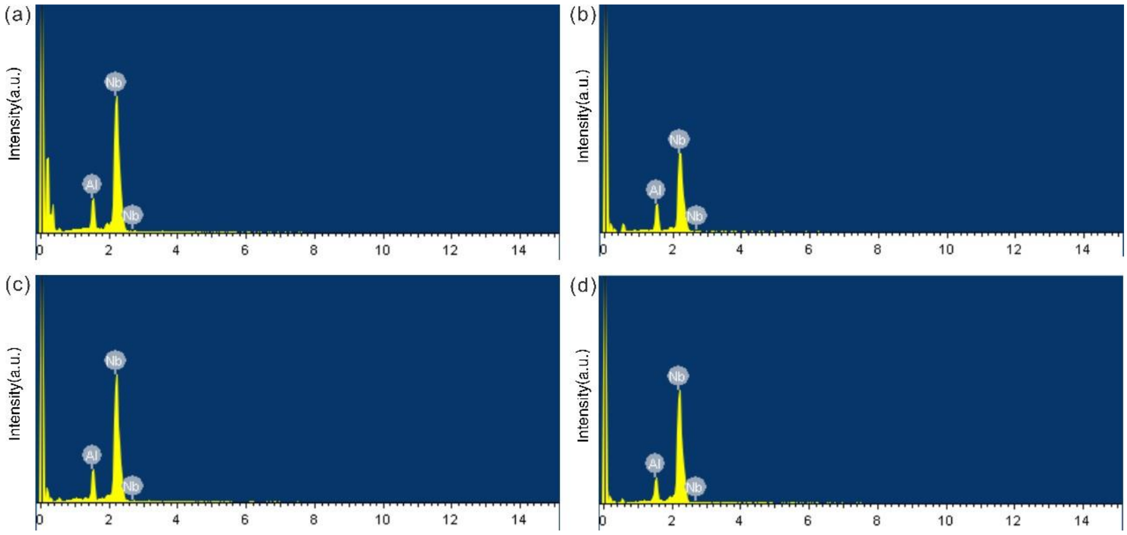

Figure S6 EDS images of Nb_3Al samples sintered at 1400°C for 10h with Nb_p: Al= (a) 2.9:1; (b)3.0:1; (c)3.5:1; (d) 3.75:1

S4. Crystal data of Nb [2]

Space group: $Im\bar{3}m$

Lattice parameter： a = 3.306 Å

Wyckoff position and atomic position: Nb 2a, (0, 0, 0)

S5. Particle Models and Calculated Results

To understand the face, size, and shape dependence of MSE for a particle, we have designed four families of models as shown in Figure S5. Family a) contains five models built by expanding the basic unit cell of BCC Nb along the b axis, i.e. [010] direction ([uvw] indices of a lattice direction; $< \mathrm{uvw} >$ indices of a set of all symmetrically equivalent lattice directions; {hkl} indices of a set of all symmetrically equivalent crystal faces), each step by one unit cell length; while family b) contains ones built by a similar way as for type a) along three equivalent lattice directions simultaneously $< 100 >$; family c) are ones built by expanding the BCC structure of Nb from a center atom along the 12 equivalent lattice directions $< 110 >$, each step by one inter-planar spacing d_{110} ; family d) are similarly built as type c) along the eight equivalent $< 111 >$ directions. On the left side of Figure 4 in the main text show the starting models in each type, respectively, while on the right side the second models in each type are given, respectively. In each type, we have prepared 5 models as shown in Figure 4. To calculate E_{model}^{N} in Eq. (4) on the same footing as E_{bulk}^{N}, a 3D periodic lattice model with a vacuum layer of 15 Å between the models is used. The calculated energies were used as input to calculate the MSEs, E_S, E_N, by Eq. (3) and (4), respectively. The calculated results are shown in **Table S1.**

Table S1. Total energy of a particle model with N atoms, total energy of N atoms in a bulk solid without surface and the average surface energy normalized to one atom of different models

series	structure	N	E_{Model}^{N}(eV)	$N \cdot \tilde{E}_{bulk}$(eV)	E_N(eV/atom)
	1×1×1	9	-55.173857	-90.6323445	3.939831944
	1×2×1	14	-92.564155	-140.983647	3.458535143
line	1×3×1	19	-130.42635	-191.3349495	3.205715763
	1×4×1	24	-167.9992	-241.686252	3.070293833
	1×5×1	29	-205.61143	-292.0375545	2.98021119
	1×1×1	9	-55.173857	-90.6323445	3.939831944
	2×2×2	35	-267.26019	-352.4591175	2.434255071
{100}	3×3×3	91	-768.05242	-916.3937055	1.630124016
	4×4×4	189	-1676.4459	-1903.279235	1.200176373
	5×5×5	341	-3115.8097	-3433.958831	0.932988652
	{110}-1	15	-120.45307	-151.0539075	2.040055833
	{110}-2	65	-576.80277	-654.5669325	1.196371731
{110}	{110}-3	175	-1623.3315	-1762.295588	0.7940805
	{110}-4	369	-3502.5733	-3715.926125	0.578191936
	{110}-5	671	-6461.9671	-6757.144796	0.439907147

{111}	{111}-1	7	-23.861282	-70.4918235	6.661505929
	{111}-2	33	-232.26458	-332.3185965	3.031939894
	{111}-3	95	-781.10391	-956.6747475	1.848114079
	{111}-4	209	-1836.1826	-2104.684445	1.284697821
	{111}-5	391	-3781.0417	-3937.471856	0.400077124

Table S2. The calculated data used to calculate the $E_{S.}$ The surface area *S* in the third column corresponds to the *N*-atom particle in Table S1. The E_{slab}^{N} in Eq. (3) of the main text is replaced by the E_{Model}^{N}.

series	structure	S (Å2)	E_{Model}^{N} (eV)	$N \cdot \tilde{E}_{bulk}$(eV）	E_S(eV/Å2)
line	1×1×1	65.58971814	-55.173857	-90.6323445	0.270305229
	1×2×1	109.3161969	-92.564155	-140.983647	0.221465315
	1×3×1	153.0426757	-130.42635	-191.3349495	0.198992207
	1×4×1	196.7691544	-167.9992	-241.686252	0.187242386
	1×5×1	240.4956332	-205.61143	-292.0375545	0.179683355
{100}	1×1×1	65.58971814	-55.173857	-90.6323445	0.270305229
	2×2×2	262.3588726	-267.26019	-352.4591175	0.162370967
	3×3×3	590.3074633	-768.05242	-916.3937055	0.125647476
	4×4×4	1049.43549	-1676.4459	-1903.279235	0.108073977
	5×5×5	1639.742954	-3115.8097	-3433.958831	0.097011891
{110}	{110}-1	92.757784	-120.45307	-151.0539075	0.16495024
	{110}-2	371.0315312	-576.80277	-654.5669325	0.104794547
	{110}-3	834.818267	-1623.3315	-1762.295588	0.083230143
	{110}-4	1484.129299	-3502.5733	-3715.926125	0.071878112
	{110}-5	2318.9466	-6461.9671	-6757.144796	0.063644781
{111}	{111}-1	75.736483	-23.861282	-70.4918235	0.307847286

	{111}-2	302.9459314	-232.26458	-332.3185965	0.165135105
	{111}-3	681.628346	-781.10391	-956.6747475	0.128787805
	{111}-4	1211.783726	-1836.1826	-2104.684445	0.110787857
	{111}-5	1893.412071	-3781.0417	-3937.471856	0.09947968

References

1. Munitz, A.; Gokhale, A.B.; Abbaschian, R. The effect of supercooling on the microstructure of Al-Nb alloys. *J. Mater. Sci.* **2000**, *35*, 2263–2271. https://doi.org/10.1023/A:1004783011253
2. Roberge, R. Lattice-parameter of niobium between 4.2 and 300 k. *J. Less-Common Met.* **1975**, *40*, 161–164.
